# The effect of inhibition of PP1 and TNFα signaling on pathogenesis of SARS coronavirus

**DOI:** 10.1186/s12918-016-0336-6

**Published:** 2016-09-23

**Authors:** Jason E. McDermott, Hugh D. Mitchell, Lisa E. Gralinski, Amie J. Eisfeld, Laurence Josset, Armand Bankhead, Gabriele Neumann, Susan C. Tilton, Alexandra Schäfer, Chengjun Li, Shufang Fan, Shannon McWeeney, Ralph S. Baric, Michael G. Katze, Katrina M. Waters

**Affiliations:** 1 Computational Biology and Bioinformatics Group, Pacific Northwest National Laboratory, Richland, WA 99354 USA; 2 Department of Epidemiology, University of North Carolina Chapel Hill, Chapel Hill, NC 27599 USA; 3 Department of Pathobiological Sciences, School of Veterinary Medicine, Influenza Research Institute, University of Wisconsin-Madison, Madison, WI 53715 USA; 4 Division of Biostatistics, Department of Public Health and Preventive Medicine, Oregon Health and Science University, Portland, OR 97239 USA; 5 Knight Cancer Institute, Oregon Health and Science University, Portland, OR 97239 USA; 6 Department of Microbiology, University of Washington, Seattle, WA 98195 USA; 7 Department of Microbiology and Immunology, University of North Carolina at Chapel Hill, Chapel Hill, NC 27599 USA

**Keywords:** Systems biology, SARS coronavirus, Pathogenicity, Network

## Abstract

**Background:**

The complex interplay between viral replication and host immune response during infection remains poorly understood. While many viruses are known to employ anti-immune strategies to facilitate their replication, highly pathogenic virus infections can also cause an excessive immune response that exacerbates, rather than reduces pathogenicity. To investigate this dichotomy in severe acute respiratory syndrome coronavirus (SARS-CoV), we developed a transcriptional network model of SARS-CoV infection in mice and used the model to prioritize candidate regulatory targets for further investigation.

**Results:**

We validated our predictions in 18 different knockout (KO) mouse strains, showing that network topology provides significant predictive power to identify genes that are important for viral infection. We identified a novel player in the immune response to virus infection, Kepi, an inhibitory subunit of the protein phosphatase 1 (PP1) complex, which protects against SARS-CoV pathogenesis. We also found that receptors for the proinflammatory cytokine tumor necrosis factor alpha (TNFα) promote pathogenesis, presumably through excessive inflammation.

**Conclusions:**

The current study provides validation of network modeling approaches for identifying important players in virus infection pathogenesis, and a step forward in understanding the host response to an important infectious disease. The results presented here suggest the role of Kepi in the host response to SARS-CoV, as well as inflammatory activity driving pathogenesis through TNFα signaling in SARS-CoV infections. Though we have reported the utility of this approach in bacterial and cell culture studies previously, this is the first comprehensive study to confirm that network topology can be used to predict phenotypes in mice with experimental validation.

**Electronic supplementary material:**

The online version of this article (doi:10.1186/s12918-016-0336-6) contains supplementary material, which is available to authorized users.

## Background

The emergence of Severe Acute Respiratory Syndrome Coronavirus (SARS-CoV), and more recently Middle East Respiratory Syndrome Coronavirus demonstrate the threat of coronavirus zoonosis to human health and the global economy [1–3]. SARS-CoV is a positive stranded RNA virus that emerged in 2002 and 2003 in Guangdong Province, China likely from a pool of closely related coronaviruses that circulate in horseshoe bats [4]. Infected patients typically presented with fever and evidence of respiratory illness, general malaise and lower respiratory tract symptoms including cough and shortness of breath, and had an overall fatality rate of approximately 10 % [5]. Despite this threat we are poorly prepared to develop rapid strategies to ameliorate coronavirus disease severity in an outbreak setting.

Viral pathogenesis has been extensively studied for decades, yet the root causes remain poorly understood. Furthermore, high mutation rates of RNA viruses allow them to quickly adapt to changes in their host environment resulting in a complex system of virus and host restriction factor evolution [6]. While several endpoints of viral infection can be measured in animal models of disease (e.g., viral replication, immune cell infiltration, body weight loss, time to death), the molecular and cellular mechanisms that determine the severity of these effects are not well-resolved. However, high throughput molecular approaches offer a way to discover novel host response genes, proteins, and pathways that contribute to the systems-level development of pathogenesis.

A key tool of systems biology, network-based strategies can provide contextual information about broad spectrum, druggable targets, such as host regulatory proteins that drive the critical functional responses comprising the pathobiology of these viruses [7]. Network-based methods have been used to identify critical regulatory nodes within signaling networks and produce mathematical models of disease processes [8–12]. Analysis of coexpression-type networks has been used previously to identify genes and proteins of increased importance to controlling system-wide host responses [10, 12–15]. The topological locations of genes in these networks can be used to identify hubs, genes that are connected to many other genes and therefore may be exerting a regulatory influence, and topological bottlenecks, genes that bridge disparate sub-networks and may serve as mediators of transitions between system states [16–18]. Recently, a node’s participation in network motifs in directional networks was shown to be an effective predictor of importance [19]. However, directional interaction networks are not always available for a given system, making methods for studying non-directional networks of interest. While topology-based approaches have been validated for in vitro systems, it is not clear that topology of co-expression networks derived from complex tissues with multiple cell types will be effective in prediction of whole-organism phenotypes. Additionally, little if any systematic experimental validation of network-based predictions made from whole organism studies has been undertaken.

Previously, we published time course studies of SARS-CoV infection in mice, gathering transcriptomic data from multiple time points and doses. We analyzed these data to identify critical targets using weighted gene correlation network analysis (WGCNA), an approach which groups similarly behaving genes into modules, then identifies genes with the most representative expression behavior within each module [20]. Here we select additional targets using the previous dataset and determine their role in SARS-CoV infection in mice. Weight loss phenotypes from infections in selected null mice were examined in new experiments reported here as well as from previously published studies. We identified a novel player in the immune response to virus infection, Kepi, an inhibitory subunit of the PP1 complex, which protects against SARS-CoV pathogenesis. We also found that receptors for the proinflammatory cytokine, TNFα, promote pathogenesis, presumably through excessive inflammation. Our results reveal new insight into the critical balance between over- and under-stimulating the innate immune response to infection. We validated several network-based approaches systematically using multiple KO mouse strains from this and previous studies, and found that ranking genes based on their network topology makes even better predictions of effect on pathogenesis than does WGCNA or simple differential expression. This study represents a critical step toward the validation of computational modeling approaches which can explain the mechanisms underlying changes in pathogenesis and predict regulators critical to this process. This is the first instance of confirmation that network topology can be used to predict phenotypes in mice with experimental validation. Also, the confirmed role of TNFα receptors and the new role of Kepi are novel contributions to SARS-CoV literature.

## Results

### Generation of transcriptomic network models

To generate networks representing host response to viral infection, we analyzed the transcriptional response to SARS-CoV infection from a recently published study [20]. In the previous study 20-week-old C57BL/6 J mice were mock-infected or infected with SARS-CoV at 10^2^, 10^3^, 10^4^, and 10^5^ plaque forming units (PFU). Lung tissues were collected at 1, 2, 4, and 7 days post-infection (DPI) for microarray analysis. Mice infected with the highest dose experienced severe weight loss and either succumbed to infection or required humane euthanasia [20, 21]. Mice infected with all other doses experienced transient weight loss and recovered from infection. We used the transcriptomic data from this experiment to generate modules using the weighted gene correlation network analysis (WGCNA), which establishes groups or modules of genes representing the main expression patterns in the process being studied [20]. WGCNA identifies genes that are highly central to modules (groups of similarly expressed genes), thus having high module centrality scores (K_ME_); these genes are postulated to play an important role in overall function such as pathogenesis. We used this approach in the previous study to identify Serpine1 as important for SARS-CoV pathogenesis.

This approach identifies genes that are related to each other by expression pattern, but not those that are highly central to the complete system. To address this we generated an association network to identify topological bottlenecks, which are genes whose expression patterns are similar to those of two groups of co-expressed genes, and thus form a link between these modules in the network. We first constructed a network of genes related by mutual information in expression patterns over time using the context likelihood of relatedness (CLR) method [22], as we have done previously to identify regulatory relationships, functional associations, or simply coordinated behavior [13, 23, 24]. Similar to protein-protein interaction networks, we have found that topological bottlenecks within transcriptional networks are significantly enriched in genes that have particular importance to the systems-level phenotypes being studied in the experiments [12, 23–27]. In the current study, genes identified as topological hubs and bottlenecks are predicted to play important roles in regulating the host response to viral infection, and may affect virus-induced disease severity.

We hypothesized that, in general, centrality in association networks and gene expression modules could identify important components of the host response to SARS-CoV.

### Assessment of network predictive power

To examine this hypothesis we ranked all genes in our transcriptional network using betweenness centrality [28] and degree centrality, as well as K_me_ values from WGCNA analysis [20]. To evaluate the performance of the rankings, we gathered a set of 11 previously published SARS-CoV infection studies [20, 29, 30] with KO mouse strains (Table 1). We considered studies where weight loss was used as a measure of pathogenicity.

**Table 1:** Summary of targets validated

Rank	Network Bottleneck	Network hub	WGCNA	Differential expression**
1	*Stat1	*Ccr5	*Myd88	Cxcr3
2	*Ccr5	*Myd88	*Ccr5	*Ccr5
3	*Myd88	*Stat1	Cxcr3	*Serpine1
4	*Kepi	*Kepi	*Stat1	*Ccr1
5	*Serpine1	Cxcr3	*Kepi	Ido1
6	*Ccr1	Ido1	Ido1	Plat
7	Cxcr3	*Ccr1	Plat	*Stat1
8	Plat	*Serpine1	*Serpine1	Il1r1
9	Ido1	Il28ra	Il28ra	Il18r1
10	Ptges2	Plat	*Ccr1	*Ccr2
11	Il1r1	*Ccr2	*Ccr2	*Myd88
12	Il28ra	Ptges2	Il18r1	Ifnar
13	*Ccr2	Il1r1	Ptges2	Il28ra
14	Ifnar	Il18r1	Il1r1	Ptges2
15	Il18r1	Ifnar	Ifnar	*Kepi

Asterisks(*) designate null mice with altered weight loss phenotypes. Each column represents a ranking of the genes using the indicated metric. Ccr1, Ccr2, Ccr5, Myd88, Il18r1, and Il1r1 assessed in Sheahan et al. 2008 [30]. Stat1, Ifnar, and IL28ra assessed in Frieman et al. 2010 [29]. Serpine1 and Plat assessed in Gralinski etal. 2013 [20]. All others assessed in the current study. For Stat1 and Ifnar, background strain used was 129. For Il28ra, background strain used was Balb/c. **Ranked by absolute differential expression versus mock at day 1 post-infection

Using the compiled weight loss data, we evaluated different ranking approaches for their ability to predict phenotypic outcome. We assessed the ability of the individual topological ranks (bottlenecks using betweenness and hubs using degree centrality and K_me_) to classify genes as to their pathogenesis phenotypes in KO mice. We also included differential expression, a standard method for predicting gene importance, from day 1 post-infection. Assessment was performed using a receiver-operator characteristic (ROC) curve, which takes into account the levels of false positive and false negative predictions at the same time without the need to place an arbitrary threshold for the ranking. The area under the ROC curve (AUC) will be 1.0 when the method perfectly classifies the examples with no false positive or false negative predictions, and it will be 0.5 for rankings that are equivalent to random choice of examples.

This assessment revealed that network measures could predict phenotype very well yielding ROC AUCs of 0.9, 0.93, and 0.83 for betweenness centrality, degree centrality, and WGCNA K_me_, respectively. Differential expression after infection performed slightly worse than network measures, giving an ROC AUC of 0.77. Though these results were promising we wanted to validate the approach on novel predictions to further characterize the method.

### Target selection and validation

We therefore followed up on more candidates by conducting SARS-CoV infections in null mice. In the previous study, a single WGCNA module (and its associated genes) was selected for follow-up study based on its unique properties. For the current study, we selected KO target genes based on various criteria. Tnfrsf1b and Kepi had high (96.9) and moderately high (85.8) percentile scores for network degree centrality, respectively. In addition, given the fact that Tnfrsf1b and Tnfrsf1a constitute the primary receptor for TNFα as a heterocomplex, we included the *Tnfrsf1a/1b*^*−/−*^ double KO as well. Cxcr3, Ido1, and Ptgs2 were also selected based on prior interest in identifying critical mediators of the immune/inflammatory response not previously known to influence SARS-CoV infection. Importantly, all choices were heavily influenced by KO mouse availability. We reasoned that allowing KO availability to influence target selection (instead of choosing candidates at the absolute top of network rankings) was a reasonable approach, since network-based scores are not expected to rank genes in the precise order of their level of impact on biological processes, but are rather likely to position genes in approximate rankings of importance. Additional file 1 shows the network degree centrality scores for the selected genes, which fall across a range of values due to the various criteria used to select them.

Groups of mice were infected with SARS-CoV and assessed for weight loss over a seven-day period along with appropriate wild type control infected mice, similar to previously published studies [20, 29, 30]. Titer and weight loss for these mutants are provided in Additional file 2. For each experiment we determined whether the null mouse had a significantly altered phenotype relative to wild type as assessed by weight loss. Though this may be an imperfect measure of pathogenesis it is an accepted method that has been utilized broadly [20, 29, 30], and importantly in the studies we used to validate our network method. Because the combined previous and current experiments provided data for genes occupying a wide range of network score values, we assessed the effectiveness of network betweenness, network degree centrality, and WGCNA analysis in identifying genes relevant to SARS-CoV infection. Thus our assessment considers whether network topology can discriminate between presence/absence of phenotype (Table 1).

**Fig. 1:**
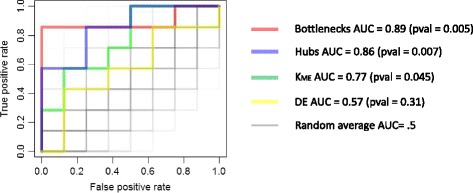
Topological rankings work better to predict mouse phenotype than differential expression or expert selection. The ability of each method to correctly classify genes as having a significant effect on pathogenesis as determined by weight loss different than wild-type mice infected with SARS-CoV (see Table 1) was assessed using a receiver-operator characteristic curve (ROC). The area under the curve (AUC) is shown in the legend. The differential expression (DE) category indicates the range of AUC values obtained when genes were ranked by DE from all viral dose and day post-infection combinations

The results of performing an ROC analysis on the combined set of published and novel targets (Fig. 1) show a clear ability of network approaches to accurately classify pathogenesis phenotypes of null mutants as compared to random classification, recapitulating our results based on previously published null mouse infections. In comparison, differential expression ranking performed worse with the addition of our new targets with an AUC of 0.59, compared to 0.77 considering only the previously published results. While degree centrality was originally used to select some of the novel targets, our assessment shows that betweenness centrality works at least as well. Because of the inclusion of genes from all portions of the ranking (not just our top predictions), we demonstrate the value of the network topology approach to predict phenotype and identify mechanisms for pharmacological intervention of viral infections.

Since the effect of perturbing TNFR was only observed with the double-KO (see below), the individual scores of the two synergistic genes were judged to be non-meaningful for this analysis; thus we removed TNFR-null mouse strains from our ranking performance assessment. This points out a limitation of the analysis for treatment of closely interacting individual genes, and suggests that network analysis methods to handle this kind of redundancy are needed.

### Kepi and TNFα play opposing roles in pathogenesis

**Fig. 2:**
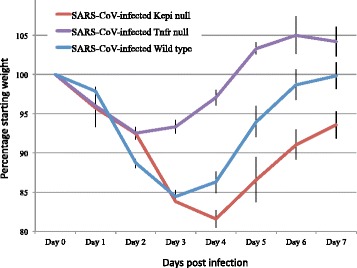
Kepi and TNFRs exhibit opposing effects on pathogenesis of SARS-CoV. C57BL/6 J mice lacking *Kepi* or *Tnfrsf1a/b* were infected with SARS-CoV at varying 10^3^ PFU. Weight loss expressed as the mean percentage of starting weight for five mice per time point up to 4 days post-infection, and three mice for 5–7 days post-infection is shown (*y axis*) plotted over time post-infection (*x axis*). Error bars indicate standard error of the means

We found that two targets, Kepi and TNFR had opposing effects on pathogenesis in mice. *Kepi* (gene symbol: *Ppp1r14c*), was a moderately high degree centrality gene with no previous association with viral pathogenesis. Kepi is a protein kinase C-regulated inhibitor of PP1 activity, and PP1 is an important regulator of a number of cellular processes including muscle contraction, neuronal activities, splicing of RNA, cell division, apoptosis, protein synthesis, and regulation of membrane receptors and channels [31, 32]. From our weight loss data we found that at 4 through 7 DPI the *Kepi*^*−/−*^ mice had significantly more weight loss than the wild type animals, indicating that Kepi may play a protective role against severe SARS-CoV-induced disease (Fig. 2). Uninfected *Kepi*^*−/−*^ mice showed no weight loss (data not shown). Titers from infected mice show a trend toward a modest increase in Kepi null mice, although the difference does not reach significance (Additional file 2).

The TNFα receptor 2 (Tnfrsf1b) was identified by our analysis as having high degree centrality but we found that infection of Tnfrsf1b^*−/−*^ mice had only a modest and non-significant effect on the weight loss phenotype (data not shown). TNFα has two main receptors Tnfrsf1a and Tnfrsf1b, and is a primary mediator of inflammation that has been implicated as important in response to upper respiratory virus infection [33–35]. Accordingly, we also investigated the response of double-null *Tnfrsf1a/1b*^*−/−*^ mice to infection with SARS-CoV. As can be seen in Fig. 2, the double-null mouse is significantly protected from weight loss associated with infection, indicating that TNFα may promote pathogenesis in SARS-CoV infection through two redundant receptors.

Though the effects on weight loss in these mutant mice were somewhat modest, the results were consistent and repeatable across the five mice tested for each target, providing a reasonable assessment of statistical significance for the results obtained.

### Validation of network model structure

**Fig. 3:**
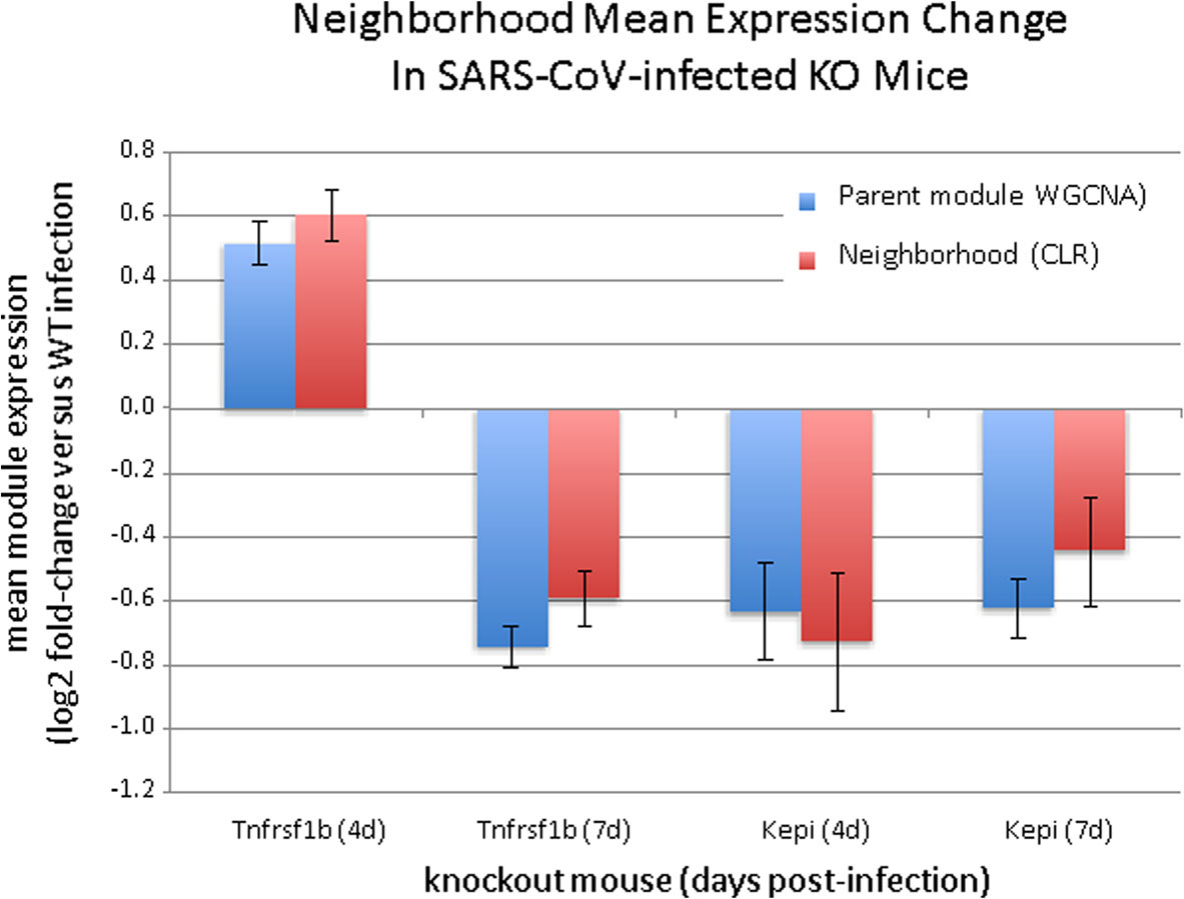
Validation of network predictions. Network neighborhoods for each of the target gene deletions tested were assessed for their expression difference from the rest of the network using a Student’s *t* test. Neighborhoods are defined in terms of the WGCNA module that contains the target gene (*blue bars*) or the first-order network of the target gene from the CLR-inferred network (*red bars*). All comparisons shown have *p* values less than 0.001. Standard error is shown for each data point as error bars. Negative mean expression indicates that deletion of the target gene is reducing the expression of its neighborhood compared to response in a wild type mouse

Our transcriptional network model identified key components of SARS-CoV pathogenicity. If our network model reflects the true structure of the underlying regulatory machinery, albeit at a lower resolution, then deletion of a target gene in an experimental system should disrupt the expression of genes adjacent to the target in the predicted network, or network “neighbors”. In order to test this hypothesis, we identified network neighbors of *Kepi* and *Tnfrsf1b* from the wild type infection studies, as well as genes co-occupying the same WGCNA modules of these genes. We then analyzed the transcriptomes of the KO mouse strains during SARS-CoV infection (see Methods) and compared the genes predicted to be altered in the KO strain (the network neighbors) with those that were actually altered by transcriptome analysis. Because our network models do not predict activation or repression effects, we can only predict that deletion of a target will have a significant effect on the expression of its direct neighbors, as compared to all other genes in the network. Figure 3 shows the expression changes in the target gene’s modules and network neighborhoods in infected KO mice. Deletion of the target genes caused predicted neighborhood genes to be significantly differentially regulated relative to infection of wild type mice (*p* values < 0.001) for all cases examined. Not only were the gene expression values of neighborhood genes significantly different from other network genes, the overlap between neighborhood genes and differentially expressed genes was significant as well (*p* < .05 by permutation test). These results support the predictions from our network models that deletion of a target gene would affect expression of those genes predicted to be downstream.

### Functional effects of Kepi and TNFR deletion on SARS-CoV pathogenesis

**Fig. 4:**
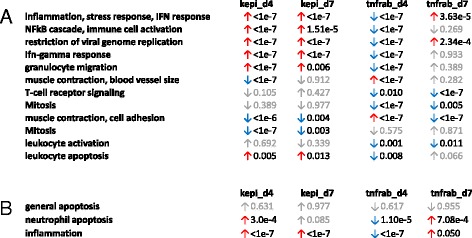
Fold changes in mut/WT for cluster categories and GO terms. **a** Expression data of mutant and WT mice from infection with SARS-CoV were analyzed using hierarchical clustering; the functional content of these clusters was determined using functional enrichment analysis. The average direction of fold change elicited by the mutant for the cluster are shown coupled with *p*-values for the significance of the change. *P*-values were calculated using permutation tests of random gene groups having identical sizes to the gene group under test. For (**b**), values were calculated as in (**a**), except genes were grouped for fold change analysis using selected GO terms instead of gene expression clusters. General apoptosis = GO:0006915, neutrophil apoptosis = GO:0001781, GO:0033029, GO:0033030, GO:0033031, GO:0006925, inflammation = GO:0006954

We anticipated that the phenotypes of the resistant *Tnfrsf1a/1b*^*−/−*^ mice and susceptible *Kepi*^*−/−*^ mice respectively would be reflected in the expression profiles of functional gene subsets, and that this information could provide insight into the mechanisms behind the observed phenotypes. Gene expression in the KO mice was examined after SARS-CoV infection and differentially expressed genes between infected wild type and KO mice were clustered based on their expression profiles and associated with functional gene ontology (GO) categories arising from enrichment analysis (Fig. 4a; average fold changes for each cluster are provided in Additional file 3). A heatmap of gene fold change values for the indicated clusters is provided in Additional file 4. The marked increase in pathogenesis we observed in the *Kepi*^*−/−*^ mice was accompanied by modest increases (relative to infection of wild type mice) across most immune response-related clusters (only clusters with discernible functionality are shown). The *Tnfrsf1a/1b* deletion showed a somewhat opposite decrease for most clusters at day 4, with this trend resolving or partially reversing at day 7. Previous studies with various influenza strains found that increases in pro-inflammatory processes were correlated with increased levels of pathogenesis [36]. Our results for *Tnfrsf1a/1b* seem to agree with this finding, since day 4 data reveals that the inflammation-related cluster is significantly down-regulated. Although day 7 shows a partial reversal of this effect, the infection is largely resolved at this point and therefore this reversal is unrelated to pathogenicity. Interestingly, the *Kepi*^*−/−*^ demonstrated an increase in expression for genes in the same cluster. It could therefore be surmised that the increased pathogenicity in the *Kepi*^*−/−*^ is a direct result of increased inflammatory activity due to absence of the gene. However, this is unlikely because of Kepi’s function as an inhibitor of the protein phosphatase PP1, which is known to regulate diverse cellular functions. PP1 opposes the following pro-inflammatory processes: TNFα activation, NF-kB activation [37], prostaglandin synthesis [38], neutrophil activation through inhibition of ROS generation, and promotes down-regulation of pro-inflammatory genes. Thus the observed increase in genes related to inflammatory processes may be a compensatory response caused by chronic absence of PP1 inhibition (see Discussion). PP1 has also been shown to contribute to apoptosis signaling in neutrophils [39, 40]. Accordingly, we saw a gene expression increase in the cluster associated with leukocyte apoptosis in the *Kepi*^*−/−*^ mice at both 4 and 7 DPI, suggesting that the removal of the block on PP1 causes an increase in apoptotic mechanisms. These findings were borne out by examining expression of all genes associated with the GO terms “inflammatory response”, “apoptosis”, and “neutrophil apoptosis” (Fig. 4b). Interestingly, an effect on apoptosis could only be observed when the more specific “neutrophil apoptosis” term was used. Fold changes and significance measures for genes in all clusters are provided in Additional file 5.

Neutrophils and monocytes are known to be key players in an inflammatory loop induced in influenza pathogenesis [36]. Accordingly, we examined expression profiles of genes specific to these cells in *Kepi*- and *Tnfrsf1a/1b*^*−/−*^ mutant mice. Consistent with the role of infiltrating neutrophils and monocytes in response to infection, we observed significant down-regulation of both monocyte (*p*-value = 0.00038) and neutrophil (*p*-value = 0.0036) markers in the protected *Tnfrsf1a/1b*^*−/−*^ mice at day 4 post-infection, but not at day 7 (Additional file 6). The susceptible *Kepi*^*−/−*^ mice have somewhat elevated expression of neutrophil and monocyte markers at both time points, but as discussed above and the Discussion section, this is not likely to be a direct result of Kepi loss/PP1-activation, but is likely a compensatory effect.

## Discussion

In this study we employed network-based models of host response to SARS-CoV to predict target nodes critical to the pathogenesis of infection. We make two contributions in this paper. Firstly, we demonstrate that ranking genes using unbiased network analysis provides good prediction of pathogenic phenotype in KO mice relative to levels expected by random chance. Second, our work highlights the critical balance involved in regulating inflammatory machinery during SARS-CoV infection, and suggests that inhibition of TNFα or PP1 signaling may represent viable avenues for future investigations into effective pharmaceutical therapies.

Our network models of mouse lung response to infection with SARS-CoV were based only on transcriptional data from infection of wild type mice. To demonstrate the efficacy of our approach, we compiled results of KO mice from this and other studies that tested the role of various genes in SARS-CoV infection of mice. These candidates resulted in roughly equal numbers of positive and negative outcomes, and thus represented an effective test of our approach. Target candidate identification through network analysis has been used previously, but *in silico/*in vitro validation has only occurred in cell culture and bacterial systems [14, 24, 25, 27]. We found that our modeling approach using network hub or bottleneck ranking provides significant prediction of genes important in pathogenesis. The results from this study and previous studies validated our approach showing that network approaches perform better than differential gene expression to identify important genes for pathogenesis.

We then showed that the network neighborhood predictions made by these network models were consistent with expression data derived from KO mice infected with virus (Fig. 3). Though these studies only validated a portion of the network models, this is an important step toward construction of more robust and complete models of pathogenesis, especially in relationship to how single gene KOs perturb the host signaling networks and understanding redundancy.

It is clear however, that improvements in modeling will result in more mechanistic hypotheses and quantitative relationships, which are currently lacking in our approach. For example, the gene expression network developed in this study does not predict directionality, and it is clear from our transcriptional analysis of the KO mice that the effect on predicted downstream genes is not complete. Future studies can use these data to refine the network model and provide predictions of dependency and directionality.

**Fig. 5:**
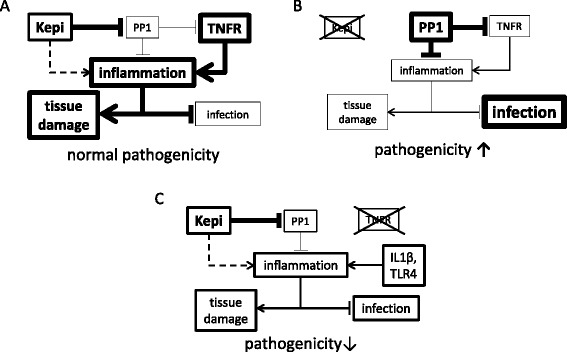
Kepi and TNFα signaling during SARS-CoV infection. Proposed model for the influence of Kepi, PP1, and TNFα signaling on SARS-CoV-mediated lung pathogenesis during WT conditions (**a**), *Kepi* deletion (**b**), and *Tnfrsf1a/1b* deletion (**c**). Bold lines indicate strong effects, thin lines indicate weak or diminished effects; dashed lines indicate indirect effects

Our results reveal opposite effects of Kepi and TNFα receptor removal during SARS-CoV infection. Figure 5 depicts possible mechanisms for the effects of removing TNFα or Kepi during SARS-CoV infection. As shown in Fig. 5a, Kepi is known to inhibit PP1, which drives apoptosis in neutrophils. In general PP1 exerts an anti-inflammatory effect on innate immune machinery, such that Kepi-mediated PP1 inhibition promotes inflammatory processes. Removing Kepi (Fig. 5b) releases the restraint on PP1, likely resulting in a general downregulation of innate immunity and decreased capacity to resist the effects of infection and increased pathogenicity. TNFα is a potent driver of leukocyte-mediated inflammation which in the case of normal SARS-CoV infection likely causes significant tissue damage. Removal of TNFRs (Fig. 5c) may disable a component of the inflammatory response, such that tissue damage is diminished. However, non-TNFα-related mechanisms (e.g. through IL1β, TLR4 etc.) could still be able to control progress of the infection, and the net result is decreased pathogenicity. Thus our studies appear to have highlighted the role of a critical balance between too little and too much inflammation in pathogenesis during murine SARS-CoV infection. Interestingly, we found that the expression of *Kepi* doubles in *Tnfrsf1a/1b*^*−/−*^mice during infection (not shown), suggesting the presence of a negative feedback loop between Kepi and TNFα signaling. While Kepi and TNFα signaling do not directly oppose each other and they are clearly not the only two important components of inflammatory regulation, study of both null mice reveals that simultaneously promoting Kepi-mediated repression of PP1 signaling and limiting TNFα-driven inflammation may lessen the pathogenic effects of SARS-CoV infection.

This balance has been demonstrated previously by showing that targeting SARS-CoV-driven inflammation through NF-kappaB signaling can alleviate SARS-CoV pathogenicity in mice [41]. Targeting inflammation during SARS through TNFα inhibition has been previously recommended based on bioinformatics analysis and expert opinion; however the current study provides the first experimental evidence for this hypothesis. In addition, since loss of Kepi results in increased pathogenesis, PP1 inhibition represents a second possibility for follow-up studies on SARS-CoV pharmaceutical treatment. Since removal of the innate PP1 inhibitor likely has the effect of crippling the immune response, exogenous inhibitor may have the desired effect of enhancing innate immunity to an optimal degree, although careful titration of therapeutic doses may be necessary to avoid an overactive immune response, and toxicity may be an issue. Inhibition of TNFα receptor signaling may represent a more promising potential therapy, since total deletion of TNFRs led to a favorable outcome. The presence of semi-redundant innate immune signaling remaining in TNFR-null mice is apparently sufficient to control SARS-CoV infection, so that signaling through these receptors can be aggressively targeted. Future studies will investigate the effects of these pharmaceutical therapies using the mouse model.

One seemingly contradictory result was the elevated expression of inflammation-related genes in *Kepi*^*−/−*^ mice. This appears surprising given the antagonistic nature of PP1 signaling toward innate immune processes. Removal of the PP1 inhibitor results in unregulated PP1 activity, which shuts down innate immune response cascades and promotes neutrophil apoptosis, although one report documented PP1 opposing apoptosis in these cells [42]. Since transcriptomics studies can only monitor mRNA transcript levels, data from protein phosphorylation states (where much of the critical signaling events may be manifested) were not collected in this study. Induction of these genes is likely an attempt to augment immune response signaling in the face of unchecked PP1. The slight increases in neutrophil- and monocyte-specific genes in the *Kepi*^*−/−*^ mice may suggest that rather than decreasing levels of neutrophils through apoptosis, PP1 is shutting down inflammatory pathways within these cells in mutant mice. Thus while gene expression is increased, phosphatase activity may still negate much of the intended signaling response to infection.

Interestingly, removal of several other components of the immune response in previous work also resulted in susceptible phenotypes. Deletion of chemokines and receptors important for neutrophil and monocyte recruitment and chemotaxis (Ccr1, Ccr2, and Ccr5) all increased pathogenesis (Table 1) [30] and lung pathology, indicating that these genes play an essential role in protecting the host from SARS-CoV pathogenesis. Deletion of *Ifnar*, which plays an important role in antiviral effects, did not have a significant effect on pathogenesis of SARS-CoV [29], likely due to the number of interferon antagonists encoded in the SARS-CoV genome. This was also true of the cytokine receptor Il1r1, which is a mediator of inflammation, and Cxcr3, a chemokine receptor implicated in neutrophil involvement in ARDS [43]. Given these observations and the somewhat modest effects on weight loss exhibited in our study, the effects of *Kepi* and *Tnfrsf1a/1b* on pathogenesis are likely to be part of a larger picture involving inflammation and their roles will require further investigation.

## Conclusions

The current study provides 1) validation of network modeling approaches for identifying important players in virus infection pathogenesis, and 2) a step forward in understanding the host response to an important infectious disease. The results presented here suggest the role of Kepi in the host response to SARS-CoV, as well as inflammatory activity driving pathogenesis through TNFα signaling in SARS-CoV infections. These results will be further investigated in future studies, which will include testing of pharmaceutical compounds. Though we have reported the utility of the approach in bacterial and cell culture studies previously, this is the first comprehensive study to confirm that network topology can be used to predict phenotypes in mice with experimental validation. We envision that our approach could be used to complement traditional approaches and provide more resolution between cause and effect from large, costly systems biology studies.

## Methods

### Viruses

SARS-CoV MA15 (referred to as SARS-CoV throughout the text) was described in [44]. All experiments using live virus were performed in an animal biosafety level 3 (BSL3) containment laboratory at the University of North Carolina.

### Virus titration of mouse lung tissues

Harvested lung tissues were frozen at −80 °C in 1 mL PBS. At the time of titration tissues were thawed, homogenized for 60 s at 6000 rpm and plated in serial dilutions on Vero cells as described previously.

### RNA extraction from lung tissues and microarray analysis

Harvested lung tissues were immediately placed in 1 mL of RNAlater (Life Technologies), incubated at 4 °C overnight, and then placed at −80 °C. Later, tissues were thawed and homogenized/virus-inactivated in 1 mL of TRIzol (Life Technologies) using a tissue homogenizer (− Magnalyser, Roche). Using Agilent mouse whole genome oligonucleotide (4×44K) microarrays, microarray processing, data acquisition, quality control and differential expression analysis were similar to the experiments described [45]. Four to five mice per time point and dose were analyzed, depending on number of surviving mice. Replicate probes were summarized as mean expression and fold-change relative to time-matched mock infections were calculated using mean expression from biological replicates.

### Identification and ranking of topological bottlenecks

Microarray data was normalized using RMA [46]. Wild type virus infected data was expressed as a log2 fold-change ratio from the time-matched control sample and significantly changed genes (Student’s *t* test *p*-value < 0.05, fold-change > 1.5) were used for network inference. This yielded 8787 genes for SARS-CoV. Four additional genes targeted for KO studies were included in the network analysis so they could receive scores and rankings. The Context Likelihood of Relatedness method (CLR) was used to infer relationships between genes by assessing the mutual information between expression profiles for all pairs of genes considered, then normalizing across all relationships for the pair. After applying a CLR score cutoff of 1.5, the final network had 4697079 edges. Betweenness and degree centrality was calculated as previously described [12].

### Mouse validation experiments

*Kepi* (*Ppp1r14c* strain 013041), *Ido1* (strain 005867), *Tnfrsf1b* (strain 002620) and *Tnfrsf1a/1b* (strain 003243), *Tnfrsf1a* (strain 002818), *Ptges2* (strain 009135) and *Cxcr3* (strain 005796) KO mice along with appropriate controls were purchased from Jackson laboratories and infected at 10 weeks of age. Weight loss was assessed at each time point for 5 mice (KO and WT controls). Groups of 3 mice were harvested for each strain and time point for titration and transcriptomic analysis. Mice were lightly anesthetized with ketamine/xylazine and infected with 10^5^ PFU of SARS-CoV in a volume of 50 mL or given PBS for a mock infection. All animals were given food and water ad libitum and weighed daily. Housing and husbandry was in accordance with UNC IACUC protocols. All KO mice tested were in C57BL/6 background, which is somewhat less sensitive to SARS-CoV infection than BALB/c used in some other studies.

### Clustering and functional enrichment

Gene expression data from KO mice and corresponding controls were processed as described above. KO mice included *Ppp1r14c*, *Tnfrsf1a*, *Tnfrsf1b*, and *Tnfrsf1a/1b*, as well as *Cxcr3.* Clusters were detected using the hclust package in R for hierarchical clustering. Genes from individual clusters were submitted to enrichment analysis to identify statistical GO term enrichment using the GOstats package in R. A reciprocal procedure was also followed in which all genes matching a particular GO term were assessed for their combined transcriptional response to infection.

### Identification and ranking of eigengenes

Initial Weighted Gene Correlation Network Analysis (WGCNA) was performed as described. The analysis was performed a second time for this study, utilizing the same 8787 differentially expressed genes with the addition of 4 KO-targeted genes so that all genes targeted for KO could receive a score. Module centrality (K_ME_) was calculated as correlation with the module eigengene as previously described [20].

### GEO accessions

The wild type mouse infection transcriptomic data have been previously described and deposited in the GEO database as GSE33266. Mouse transcriptomics datasets have been deposited for the KO mice infected with SARS-CoV for *Kepi*^*−/−*^ (GSE40827), Tnfrsf1b-null (GSE40824), and Tnfrsf1a/1b-null (GSE40840) mice.

## Additional files

Additional file 1: Network degree centrality scores – degree centrality identifies the network hub genes. (XLSX 665 kb) Additional file 2: Weight loss and titer – Weight loss and titer data for various mice strains infected with SARS-CoV. (XLSX 23 kb) Additional file 3: Figure 4 fold changes – fold change data from the analysis depicted in Fig. 4. (XLSX 14 kb) Additional file 4: Companion heatmap for Fig. 4 – heatmap representing fold changes identified in Fig. 4. (PDF 249 kb) Additional file 5: Individual fold changes – individual fold change values for all genes incorporated in Fig. 4. (XLSX 1253 kb) Additional file 6: Monocyte and neutrophil marker levels – monocyte- and neutrophil-specific markers allow inference of the presence of these cells. (PDF 252 kb)

## Abbreviations

SARS-CoV: Severe acute respiratory syndrome coronavirus
MERS-CoV: Middle East respiratory syndrome coronavirus
PP1: Protein phosphatase 1
TNFα: Tumor necrosis factor alpha
TNFR: TNFα receptor
WGCNA: Weighted gene correlation network analysis
KO: Knockout
PFU: Plaque forming units
DPI: Days post-infection
K_ME_: Module centrality score
GO: Gene ontology
WT: Wild type
