# Supplementary material for: The effect of inhibition of PP1 and TNFα signaling on pathogenesis of SARS coronavirus
Source: BMC Syst Biol. 2016 Sep 23;10:93. doi: 10.1186/s12918-016-0336-6 (PMC5035469; doi:10.1186/s12918-016-0336-6)
Supplement: Additional file 4: — Companion heatmap for Fig. 4 – heatmap representing fold changes identified in Fig. 4. (PDF 249 kb) [file 12918_2016_336_MOESM4_ESM.pdf]

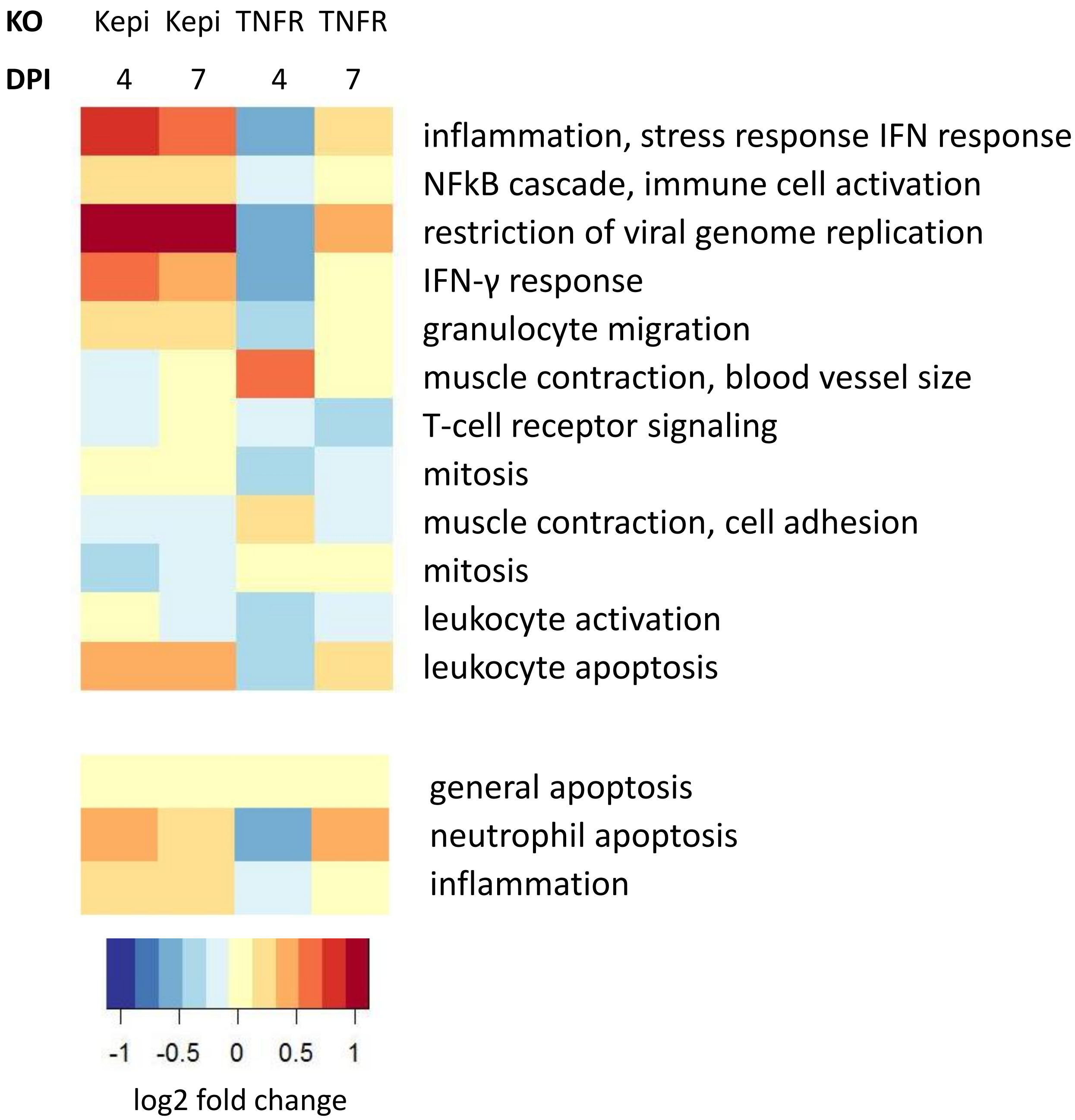


**Fold changes in mut/WT in various functional categories.** Top section: expression data of mutant and WT mice from infection with SARS-CoV were analysed using hierarchical clustering; the functional content of these clusters was determined using functional enrichment analysis (same clusters as Figure 4). Fold changes were calculated from log2 fold change in WT subtracted from log2 fold changes in KO mouse. Bottom section: Genes were grouped for fold change analysis using selected GO terms instead of gene expression clusters. KO – indicates gene targeted in null mice, DPI – days post infection.
