# Supplementary material for: The effect of inhibition of PP1 and TNFα signaling on pathogenesis of SARS coronavirus
Source: BMC Syst Biol. 2016 Sep 23;10:93. doi: 10.1186/s12918-016-0336-6 (PMC5035469; doi:10.1186/s12918-016-0336-6)
Supplement: Additional file 6: — Monocyte and neutrophil marker levels – monocyte- and neutrophil-specific markers allow inference of the presence of these cells. (PDF 252 kb) [file 12918_2016_336_MOESM6_ESM.pdf]

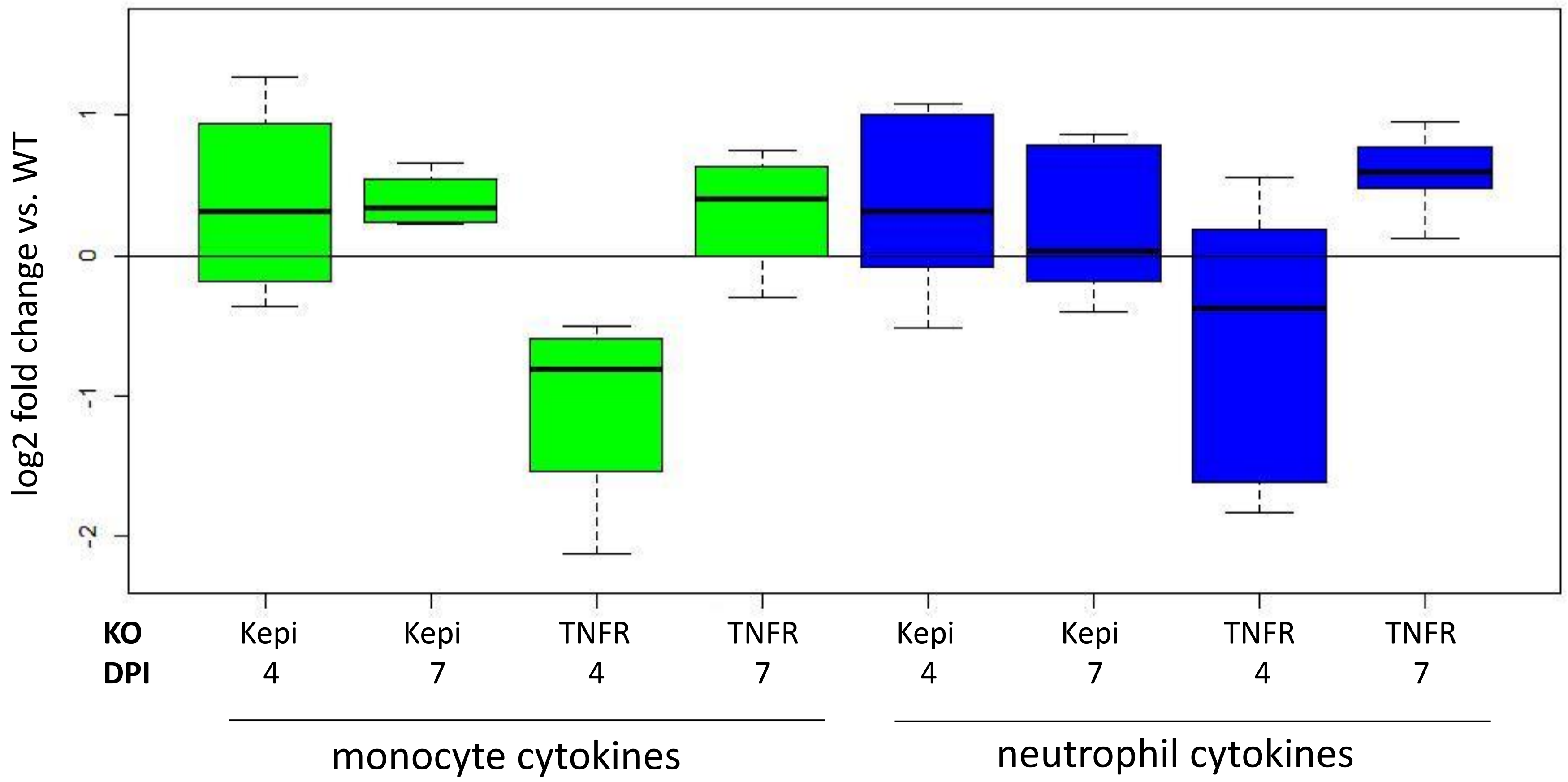

Monocyte and neutrophil chemokine expression signatures in Kepi and Tnfrsf1a/b-null mice infected with SARS-CoV. Expression of typical chemokines and receptors expressed in monocytes (Ccl2, Ccl7, Ccl12, Ccr5, Ccr1, and Cccr2) or neutrophils (Ccl7, Cxcr2, Cxcl2, Ccr1, Cxcl1, Cxcl2, Cxcl3, Ccl3, Ccl4, Ccl5) are shown as box plots for day 4 or 7 post-SARS-CoV-infection. These results indicate that the protected Tnfrsf1a/b-null mice show a significantly reduced pattern of expression associated with monocytes and neutrophils. KO – in dicates gene targeted in null mice, DPI – days post infection.
